# Effectiveness of mobile mindfulness training on stress, burnout, and work engagement of office workers: protocol for a randomized controlled trial

**DOI:** 10.3389/fpubh.2024.1440824

**Published:** 2025-01-08

**Authors:** Seung Il Lee, Seok In Yoon, Geum Ju Song, Hui Yeong Park, Sun Yong Chung, Jong Woo Kim

**Affiliations:** ^1^Department of Neuropsychiatry, College of Korean Medicine, Kyung Hee University, Seoul, Republic of Korea; ^2^Industry-Academic Cooperation Foundation, Kyung Hee University, Seoul, Republic of Korea; ^3^Department of Neuropsychiatry, Kyung Hee University Korean Medicine Hospital at Gangdong, Seoul, Republic of Korea

**Keywords:** burnout, work engagement, perceived stress, worker, smartphone application, mindfulness, protocol, randomized controlled trial

## Abstract

**Background:**

Work stress has a detrimental impact on individual health and corporate efficiency and productivity. Mindfulness reduces workers’ stress and burnout and increases work engagement and performance. Smartphone-based interventions could be an alternative to provide customized training without geographical or economic constraints. This study aims to investigate whether mobile mindfulness training (MMT) improves office workers’ stress, burnout, and work engagement.

**Methods:**

This study is a two-arm randomized controlled trial. In total, 114 office workers will be randomly assigned to one of two groups: an experimental group and a control group. The experimental group will undergo MMT, following both daily and event guidelines, for the first 4 weeks. In contrast, the control group will not receive any intervention for the first 4 weeks. During the next 4 weeks, the control group will undergo MMT for ethical reasons. Assessments will be conducted at baseline, post-intervention (fourth week), and follow-up (eighth week). The outcomes are burnout, work engagement, perceived stress, mindfulness, and vitality.

**Discussion:**

This study will serve as a basis for evaluating the effectiveness of MMT on stress, burnout, and work engagement of office workers.

**Ethics and dissemination:**

This study was approved by the Institutional Review Board of Kyung-Hee University [KHSIRB-24-063(RA)]. The results will be disseminated through peer-reviewed publications.

**Clinical trial registration:**

Identifier [KCT0009458]. https://cris.nih.go.kr/cris/search/detailSearch.do?seq=26951&status=5&seq_group=26951&search_page=M

## 1 Introduction

Work stress refers to a phenomenon in which personal factors and the workplace environment interact to negatively affect workers’ physical and psychological functions (1). Previous studies have shown that chronic stress at work can lead to physical illness and psychological disorders (2, 3). Work stress has a negative impact not only on an individual’s health but also on their attitude and performance toward their job. Work engagement refers to a state in which they highly identify with their job and are highly physically, cognitively, and emotionally involved in their job (4). Burnout, the opposite state of work engagement, appears as a response to chronic emotional and interpersonal stressors at work and results in exhaustion, cynicism, and lack of efficacy (5). In this way, chronic work stress could cause negative perceptions of the job and further worsen job performance. Indeed, previous studies have shown that excessive work-related stress reduces personal health and work motivation, increases mistakes and accidents, and ultimately reduces organizational efficiency and productivity (6, 7). According to other studies, work stress causes general health problems, increases burnout, and reduces job satisfaction and work engagement (8–10). For this reason, interest in methods for managing work stress is increasing. Common interventions for managing workers’ stress include mind–body interventions such as mindfulness and relaxation techniques (11).

Recently, various mobile healthcare applications have been developed for mental health management (12). Mobile-based interventions can deliver high-quality education without geographical and economic constraints and can provide customized education suitable for individuals (13). Furthermore, because these interventions are easy to standardize, they may be as effective in improving health outcomes as well-designed offline education (13). According to previous studies, mobile healthcare applications can reduce psychological symptoms, prevent disease, and improve wellbeing (14–16).

Mindfulness is a unique attentional ability developed through meditation practice, characterized by present-moment focus and nonjudgmental attitude. Mindfulness training is used to alleviate emotional problems and improve the quality of life in a variety of clinical groups. Previous studies have shown that mindfulness reduces stress, depression, and anxiety, and improves wellbeing (17–22). Furthermore, mindfulness training can improve workers’ mental health and job performance. According to previous studies, mindfulness reduces workers’ stress and burnout (23–26) and improves work engagement and job performance (27–29).

Because of these benefits, mindfulness has recently become a core component of several smartphone-based healthcare applications, such as Headspace and Calm. Previous studies have shown that mobile mindfulness training (MMT) reduces users’ stress, depression, and anxiety, and improves their quality of life (16, 30–33). However, most studies on the effects of mobile mindfulness training have been conducted on students or the general public, with relatively few studies targeting workers (16). Moreover, according to previous research (34), more than half of digital mindfulness-based intervention studies have been conducted with healthcare workers, while less than one-fifth of the studies have been conducted with office workers. Therefore, this study will investigate the effects of MMT on office workers’ stress, burnout, and work engagement.

This study aims to verify the effectiveness of MMT on the mental health of office workers and explore its mechanisms. The primary objective of this study is to investigate whether a four-week MMT improves office workers’ mental health, stress, burnout, and work engagement. The results of this study are predicted as follows: (i) The experimental group will exhibit reduced burnout compared to the control group after 4 weeks. (ii) The experimental group will exhibit enhanced work engagement compared to the control group after 4 weeks. (iii) The experimental group will exhibit lower perceived stress compared to the control group after 4 weeks. (iv) The experimental group will exhibit increased mindfulness compared to the control group after 4 weeks. (v) The experimental group will exhibit superior subjective vitality compared to the control group after 4 weeks.

The secondary objective of this study is to perform a follow-up examination to assess the effect of MMT on the mental health, stress, burnout, and work engagement of employees over a longer period. The results of this study are predicted as follows: (i) Burnout at follow-up will be lower than that at baseline in the experimental group. (ii) Work engagement at follow-up will be higher than that at baseline in the experimental group. (iii) Perceived stress at follow-up will be lower than that at baseline in the experimental group. (iv) Mindfulness at follow-up will be higher than that at baseline in the experimental group. (v) Subjective vitality at follow-up will be higher than that at baseline in the experimental group.

The third objective of this study is to examine differences in the effectiveness of MMT depending on the amount of application usage. The results of this study are predicted as follows: (i) The high-usage subgroup will exhibit a greater reduction in burnout compared to the low-usage subgroup after MMT. (ii) The high-usage subgroup will exhibit a greater increase in work engagement compared to the low-usage subgroup after MMT. (iii) The high-usage subgroup will exhibit a greater reduction in perceived stress compared to the low-usage subgroup after MMT. (iv) The high-usage subgroup will exhibit a greater increase in mindfulness compared to the low-usage subgroup after MMT. (v) The high-usage subgroup will exhibit a greater increase in subjective vitality compared to the low-usage subgroup after MMT.

The fourth objective of this study is to examine whether mindfulness and subjective vitality mediate the relationship between MMT and burnout or work engagement. The results of this study are predicted as follows: (i) Mindfulness will mediate the relationship between MMT and burnout. (ii) Mindfulness will mediate the relationship between MMT and work engagement. (iii) Subjective vitality will mediate the relationship between MMT and burnout. (iv) Subjective vitality will mediate the relationship between MMT and work engagement.

## 2 Methods

### 2.1 Trial design

This study is a two-arm randomized controlled trial that will investigate the effects of MMT on stress, burnout, and work engagement of office workers. Office workers aged 19 years or older who complain of job-related stress will be recruited for this study. Participants will be randomly allocated to either the experimental or control group. Participants will take part in the study for 8 weeks. The intervention will be conducted over 4 weeks. Outcome variables will be assessed at baseline (T1), post-intervention (fourth week; T2), and at follow-up (eighth week; T3).

**Figure 1:**
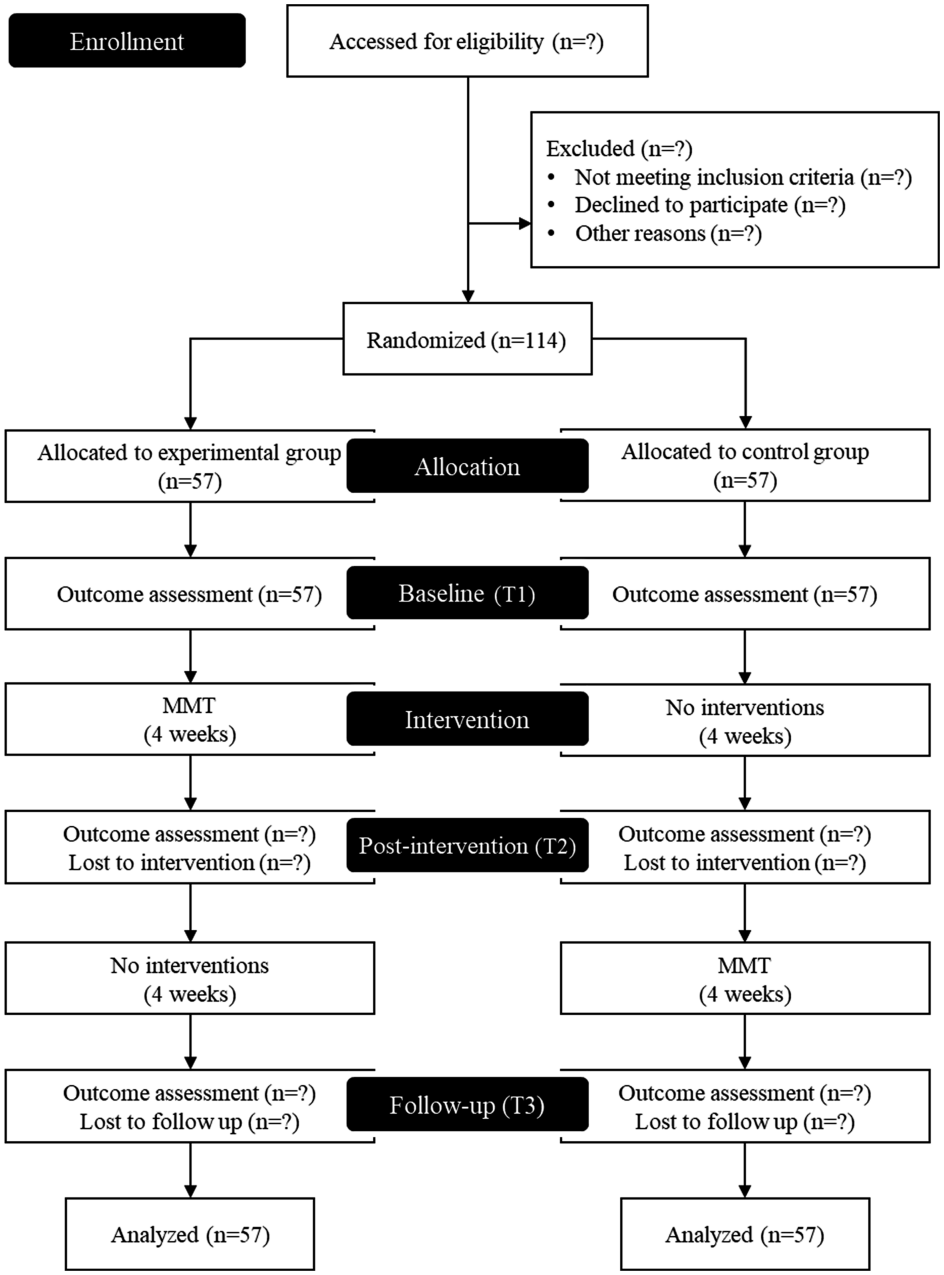
Study flow chart. MMT, mobile mindfulness training.

This trial was registered with the Clinical Research Information Service (CRIS), Republic of Korea (KCT0009458). This trial adheres to the SPIRIT guidelines and is conducted following the tenets of the Declaration of Helsinki. Figure 1 describes the flow chart of this trial design.

### 2.2 Study setting

This trial will be conducted in companies and institutions in the Republic of Korea with the approval of the Institutional Review Board of Kyung Hee University. Participants will be recruited through advertisements displayed in their workplaces. People interested in participating will be gathered at a location within their workplace and informed about the study. After receiving written informed consent, they will be screened to assess the inclusion and exclusion criteria. The intervention will be smartphone-based.

### 2.3 Eligibility criteria

#### 2.3.1 Inclusion criteria

1. Office worker over 19 years of age.
2. Participants subjectively complaining of stress related to various issues at work.
3. Participants who can participate in the study for 8 weeks.

#### 2.3.2 Exclusion criteria

1. Participants unable to use the application.

### 2.4 Interventions

#### 2.4.1 Mobile mindfulness training (MMT)

The experimental group will be provided with a mobile application called InMind for the first 4 weeks. InMind is a mental health management application developed by Demand Inc. and serviced in Korea and Japan. InMind provides mindfulness-based meditation, healing sound, and ACT-based contents (e.g., emotional diary, restructuring) for mental health, but this study used only meditation and healing sound as components of the intervention. InMind provides guided meditation, which consists of a two-week meditation curriculum for regular training and event-specific meditation for coping with specific situations. Guided meditation includes a variety of meditations such as breathing meditation, body scan, yoga, eating meditation, walking meditation, and loving-kindness meditation. Furthermore, it provides nature-based healing sounds that can help with relaxation and mindfulness.

Participants will be asked to follow both daily and event guidelines while using InMind for 4 weeks (Table 1). For the daily guidelines, participants will be asked to perform a two-week meditation curriculum consisting of 14 sessions, with participants being expected to perform one session per day. This curriculum consists of mindfulness-based training such as sitting meditation, body scan, yoga, eating meditation, and walking meditation. Each session is provided as an audio recording and is approximately 10 min long. Participants will be asked to perform the curriculum twice during the four-week intervention period.

**Table 1:** Guideline for mobile mindfulness training.

No.	Guideline	
	Event	Daily
1	In acute stress situations, select and perform one or more of the following: Concentrative meditation for quick relaxation Healing sound for relaxation.	Perform a “Two-week meditation curriculum,” one session per day. Repeat a total of 2 times for 4 weeks. *(Two-week meditation curriculum)* Session 1. Program introduction (4 min) Session 2. I want to have a deep sleep (9 min) Session 3. Breathe with nature (10 min) Session 4. Come back to breathing at any time (13 min) Session 5. Focus on the object and relax (11 min) Session 6. My happy moment (11 min) Session 7. Focus on posture and breathing (11 min) Session 8. Time to look at my mind (10 min) Session 9. Breathe comfortably and focus on your senses (12 min) Session 10. Mindfulness yoga (13 min) Session 11. Eating meditation (10 min) Session 12. Walking meditation (10 min) Session 13. Compassion meditation (13 min) Session 14. Program closure (11 min)
2	In chronic stress situations, select and perform one or more of the following: Mindfulness meditation for emotional regulation Healing sound for emotional regulation.	
3	In interpersonal stress situations, select and perform one or more of the following: Compassion meditation for improving positive emotion Healing sound for improving interpersonal relationships.	
4	In daily life stress situations, select and perform one or more of the following: Meditation that is easy to apply in daily life (e.g., meditation for deep sleep, morning meditation for a special day) Healing sound	

For event guidelines, whenever participants experience stress during their daily or work life, they will be asked to practice meditation or listen to healing sounds recommended for that stressful situation. A total of four types of stressful situations are listed, namely, acute work stress, chronic work stress, interpersonal stress, and daily life stress. In acute-stress situations, concentrative meditation and healing sounds will be recommended for quick relaxation. In chronic stress situations, mindfulness meditation and healing sounds will be recommended for emotional regulation. In situations of interpersonal stress, compassion meditation and healing sounds will be recommended to improve interpersonal relationships. In situations of daily life stress, meditation that is easy to apply in daily life (e.g., meditation for deep sleep, morning meditation for a special day) and healing sounds will be recommended. Participants will be asked to autonomously perform the recommended content for the relevant stressful situation. Education on daily and event guidelines will be provided to improve compliance with the intervention protocol.

#### 2.4.2 Wait-list control

The control group will not receive any intervention for the first 4 weeks. However, for ethical reasons, the control group will be given InMind for the next 4 weeks. The MMT procedures for the control group will be the same as that for the experimental group. The experimental group will not receive any intervention while the control group will perform MMT.

### 2.5 Discontinuation criteria

The discontinuation criteria for this study will include the following:

1. When a participant repeatedly complains of psychological or physical discomfort and wishes to drop out of the study.
2. When a participant wishes to drop out for other reasons.

### 2.6 Sample size

The minimum sample size was calculated using the G* Power program (35). A value of 0.15 indicating a small effect size and a significance level of 0.05 were set, and the power was applied as 0.80. The calculation indicated that a minimum of 90 participants is required, with a minimum of 45 participants per group. Considering a dropout rate of 20%, a maximum of 57 participants per group and a total of 114 participants will be recruited.

### 2.7 Randomization

All participants will be randomly allocated to either the experimental or the control group. A random number table will be generated by a computer and managed by the investigator (YSI). The allocation ratio between the experimental and control groups will be 1:1. Participants will be unaware of which group they have been allocated to before enrollment. The investigator (LSI) will enroll participants in the order. After enrollment, participants will be assigned to the experimental or control group based on the random number, and other investigators will be unaware of the allocation of participants.

### 2.8 Blinding

Due to the nature of the intervention, both investigators and participants cannot be blinded. However, because a standardized smartphone application is provided, the threat of bias due to investigator blinding failure may be low.

### 2.9 Assessment

Assessments are scheduled at baseline (T1), post-intervention (fourth week; T2), and follow-up (eighth week; T3). The assessment will be performed as a self-report assessment. If participants drop out, they will not be assessed further. Table 2 summarizes the assessment time points.

**Table 2:** Study assessment points.

	Screening	T1	T2	T3
Demographic information	○			
MBI-GS		○	○	○
UWES		○	○	○
PSS		○	○	○
CAMS-R		○	○	○
IVS		○	○	○

T1, baseline; T2, post-intervention; T3, follow-up; MBI-GS, Maslach burnout inventory—general survey; UWES, The Utrecht work engagement scale; PSS, Perceived stress scale; CAMS-R, Cognitive and affective mindfulness scale—revised; IVS, integrative vitality scale.

### 2.10 Primary outcomes

#### 2.10.1 Maslach burnout inventory—general survey (MBI-GS)

The Korean version of the MBI-GS will be used to assess burnout (36). This scale consists of 5 items for burnout, 4 items for cynicism, and 6 items for decreased job efficacy. Decreased job efficacy is reverse scored. The MBI-GS is a self-reported 5-point Likert scale. Each item ranges from 1 to 5. The total score ranges from 15 to 75. A higher score indicates more severe burnout. In a previous study (36), Cronbach’s alpha was 0.90 for burnout, 0.81 for cynicism, and 0.86 for decreased job efficacy.

#### 2.10.2 The Utrecht work engagement scale (UWES)

The Korean version of the UWES will be used to assess work engagement (37). This scale consists of 3 items for vigor, 3 items for dedication, and 3 items for absorption. The UWES scale is a self-reported 7-point Likert scale. Each item ranges from 0 to 6. The total score ranges from 0 to 54. A higher score indicates more immersion in one’s job. In previous studies (37), Cronbach’s alpha was 0.91 for vigor, 0.89 for dedication, and 0.90 for absorption.

### 2.11 Secondary outcomes

#### 2.11.1 Perceived stress scale (PSS)

The Korean version of the PSS will be used to assess perceived stress (38). This scale consists of 5 items for negative perception and 5 items for positive perception. Positive perception is reverse scored. The PSS is a self-reported 5-point Likert scale. Each item ranges from 1 to 5. The total score ranges from 10 to 50. A higher score indicates more perceived stress. In a previous study (38), Cronbach’s alpha was 0.77 for negative perception and 0.74 for positive perception.

#### 2.11.2 Cognitive and affective mindfulness scale-revised (CAMS-R)

The Korean version of CAMS-R will be used to assess mindfulness (39). This scale consists of 4 items for awareness, 4 items for attention, and 2 items for acceptance. CAMS-R is a self-reported 4-point Likert scale. Each item ranges from 1 to 4, and one item from the attention subfactor is reverse-scored. The total score ranges from 10 to 40. A higher score indicates a higher level of mindfulness. In a previous study (39), Cronbach’s alpha was 0.70 overall, 0.58 for awareness, 0.73 for attention, and 0.35 for acceptance.

#### 2.11.3 Integrative vitality scale (IVS)

The IVS is a self-report scale that measures physical and psychological vitality (40). The scale consists of 22 items, 11 items for physical vitality and 11 items for psychological vitality. The IVS is a self-report 5-point Likert scale. The total score ranges from 0 to 44 for physical vitality and from 0 to 44 for psychological vitality. One item (e.g., My head feels heavy and achy) is reverse scored. Higher scores indicate higher physical and psychological vitality. In a survey of 348 people, Cronbach’s alpha was 0.91 for physical vitality, 0.91 for psychological vitality, and 0.94 overall (40).

### 2.12 Data management

All collected data will be stored on the investigator’s computer and encrypted. Personal information will be replaced with a unique identifier and stored together with research information for 3 years and destroyed thereafter.

### 2.13 Statistical analyses

Analyses will be conducted according to the Intention-To-Treat (ITT) principle, and missing data will be imputed using the expectation maximization algorithm.

First, to investigate the effectiveness of MMT, a 2 (group: experimental vs. control) × 2 (survey time: T1 vs. T2) repeated-measures ANOVA will be performed for MBI-GS, UWES, PSS, CMAS-R, and IVS. If the interaction between the group and survey time is significant, a simple main effect analysis will be performed. Second, a paired *t-*test will be performed on MBI-GS, UWES, PSS, CMAS-R, and IVS in the experimental group to examine the follow-up effect of MMT. Third, a subgroup analysis will be performed to investigate the difference in effectiveness depending on the amount of application usage. For this purpose, 2 (subgroup: high-usage vs. low-usage) × 2 (survey time: baseline vs. post-intervention) repeated-measures ANOVA will be performed. Data from both the experimental and control groups will be used for subgroup analysis. If the interaction between the subgroup and survey time is significant, a simple main effect analysis will be performed. Fourth, a mediating effect analysis will be performed to investigate whether mindfulness and subjective vitality mediate the relationship between application use and burnout or work engagement.

## 3 Discussion

This study aims to investigate the effects of MMT on office workers. This study is designed as a two-armed randomized controlled trial and will primarily investigate the effects of MMT on perceived stress, burnout, work engagement, mindfulness, and subjective vitality. Additionally, this study will investigate the follow-up effect of MMT and perform subgroup analysis to compare differences in effectiveness depending on application usage. Finally, this study will investigate whether mindfulness and subjective vitality mediate the relationship between MMT and burnout or work engagement.

MMT will improve the mental health and productivity of office workers. According to previous studies, mindfulness training reduced perceived stress (16) and increased mindfulness and subjective vitality (41, 42). Mindfulness training reduced job burnout and increased job engagement among health care providers (25, 43). Furthermore, the positive effects of MMT will be maintained even after completing the intervention. According to a meta-analysis study, the follow-up effect of mindfulness meditation on anxiety and stress was found to be significant (16). These results suggest that MMT improves the mental health and productivity of office workers, and that the improved effects can be maintained even after training ends.

The amount of application usage will affect the effectiveness of MMT. According to previous research, as the number of sessions of mindfulness training increased, stress significantly decreased (44). According to a study that examined the effects of mindfulness training on a weekly basis (45), the level of mindfulness significantly increased after 2 weeks of training, and perceived stress significantly decreased after 4 weeks of training. These findings suggest that the effectiveness of MMT may also increase as the amount of application usage increases.

This study may have the following important implications. First, this study will investigate the effect of MMT on office workers’ mental health, stress, burnout, and work engagement. If the results are consistent with the hypotheses, MMT could be offered to improve individual health and productivity at work. Second, this study will investigate differences in the effectiveness of MMT depending on the amount of application usage. The results of this study will enhance our understanding of the relationship between frequency of use and the effectiveness of smartphone applications. Third, this study will investigate variables that mediate the relationship between mindfulness training and office workers’ perceived stress, burnout, and work engagement. Through this, psychological mechanisms that improve office workers’ job motivation and productivity may be verified and applied to various interventions in the future. In summary, this study has important implications that suggest a mobile-based alternative for mental health promotion and may lead to further research on effective mobile interventions.

### 3.1 Trial status

Recruitment and enrollment of participants has not started.
